# The emerging roles of UFMylation in the modulation of immune responses

**DOI:** 10.1002/ctm2.70019

**Published:** 2024-09-11

**Authors:** Zhengyan Liang, Rongxuan Ning, Zhaoxiang Wang, Xia Kong, Yubin Yan, Yafei Cai, Zhiwei He, Xin‐guang Liu, Yongkang Zou, Junzhi Zhou

**Affiliations:** ^1^ Guangdong Provincial Key Laboratory of Medical Immunology and Molecular Diagnostics School of Basic Medicine Guangdong Medical University Dongguan China; ^2^ Key Laboratory for Epigenetics of Dongguan City, China‐America Cancer Research Institute Guangdong Medical University Dongguan China; ^3^ Institute of Cancer Research Shenzhen Bay Laboratory Shenzhen China; ^4^ College of Animal Science and Technology Nanjing Agricultural University Nanjing China

**Keywords:** immune homeostasis, post‐translational modifications, UFM1, UFMylation

## Abstract

Post‐translational modification is a rite of passage for cellular functional proteins and ultimately regulate almost all aspects of life. Ubiquitin‐fold modifier 1 (UFM1) system represents a newly identified ubiquitin‐like modification system with indispensable biological functions, and the underlying biological mechanisms remain largely undiscovered. The field has recently experienced a rapid growth of research revealing that UFMylation directly or indirectly regulates multiple immune processes. Here, we summarised important advances that how UFMylation system responds to intrinsic and extrinsic stresses under certain physiological or pathological conditions and safeguards immune homeostasis, providing novel perspectives into the regulatory framework and functions of UFMylation system, and its therapeutic applications in human diseases.

## Graphical abstract

1. UFMylation regulates cGAS‐STING and RIG‐I pathways to defend pathogens.
2. UFMylation regulates the PD‐1/PD‐L1 checkpoint.
3. UFMylation component UFBP1 regulates the function of B cells.
4. UFMylation regulates inflammation‐related pathways (e.g., TLRs and NF‐κB).

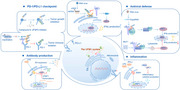

## 1 INTRODUCTION

The immune system is pivotal in maintaining physiological homeostasis by selectively eliminating invaded microorganisms and clearing aberrant or damaged cellular components.^1^ An ideal immune response eliminates potential threats, and re‐establishes homeostasis without inflicting unnecessary damage to healthy cells and tissues, while uncontrolled immune responses can be disruptive and induce tissue damage.^1^ To avoid the harmful pathology, the immune system has developed sophisticated and multilevel regulatory mechanisms. A particularly noteworthy mechanism is the post‐translational modification by ubiquitin and ubiquitin‐like molecules (UBLs). UBL modifications participate in the modulation of immune responses by specifically targeting immune receptors, adaptors, enzymes and transcriptional factors.^2^ Furthermore, UBL modifications have emerged as potential therapeutic targets for therapeutic intervention in immunological disorders.^3^ However, the roles of many other unconventional UBLs in immune responses have yet to be fully elucidated. Notably, ubiquitin‐fold modifier 1 (UFM1), first identified in 2004, stands out as an intriguing case.^4^, ^5^ The UFM1 system is highly conserved across all eukaryotic organisms, with the exception of yeast or other fungi.^4^ Over the past two decades, there have been rapid growth interests and advancements in understanding the components and functions of the UFM1 system, pointing to that the UFM1 system is crucial for the development and tissue homeostasis, and plays critical roles in cellular processes.^6^ It is not surprising that disruptions in the UFM1 system can have direct or indirect impacts on immune responses. Indeed, increasing experimental and clinical evidences suggest that defects in UFMylation are closely associated with various immune responses,^7^, ^8^, ^9^, ^10^, ^11^, ^12^, ^13^, ^14^, ^15^ leading to immune disorders and contributing to tumorigenesis.^10^, ^11^, ^16^, ^17^, ^18^, ^19^ Given the complexity of immune regulation and the potential of UFMylation as a regulatory mechanism, at present, there are no systematic reviews on the regulation of UFMylation in immune responses.

In this review, we summarise current advancements in our knowledge of the UFM1 system‐mediated mechanisms within innate and adaptive immune responses and diseases. Additionally, we assess the potential of these findings to support the development of new therapeutic interventions targeting the UFM1 system, with potential applications in inflammatory diseases, infections and even cancer therapy.

## 2 COMPONENTS AND FUNCTIONS OF UFMYLATION

**FIGURE 1:**
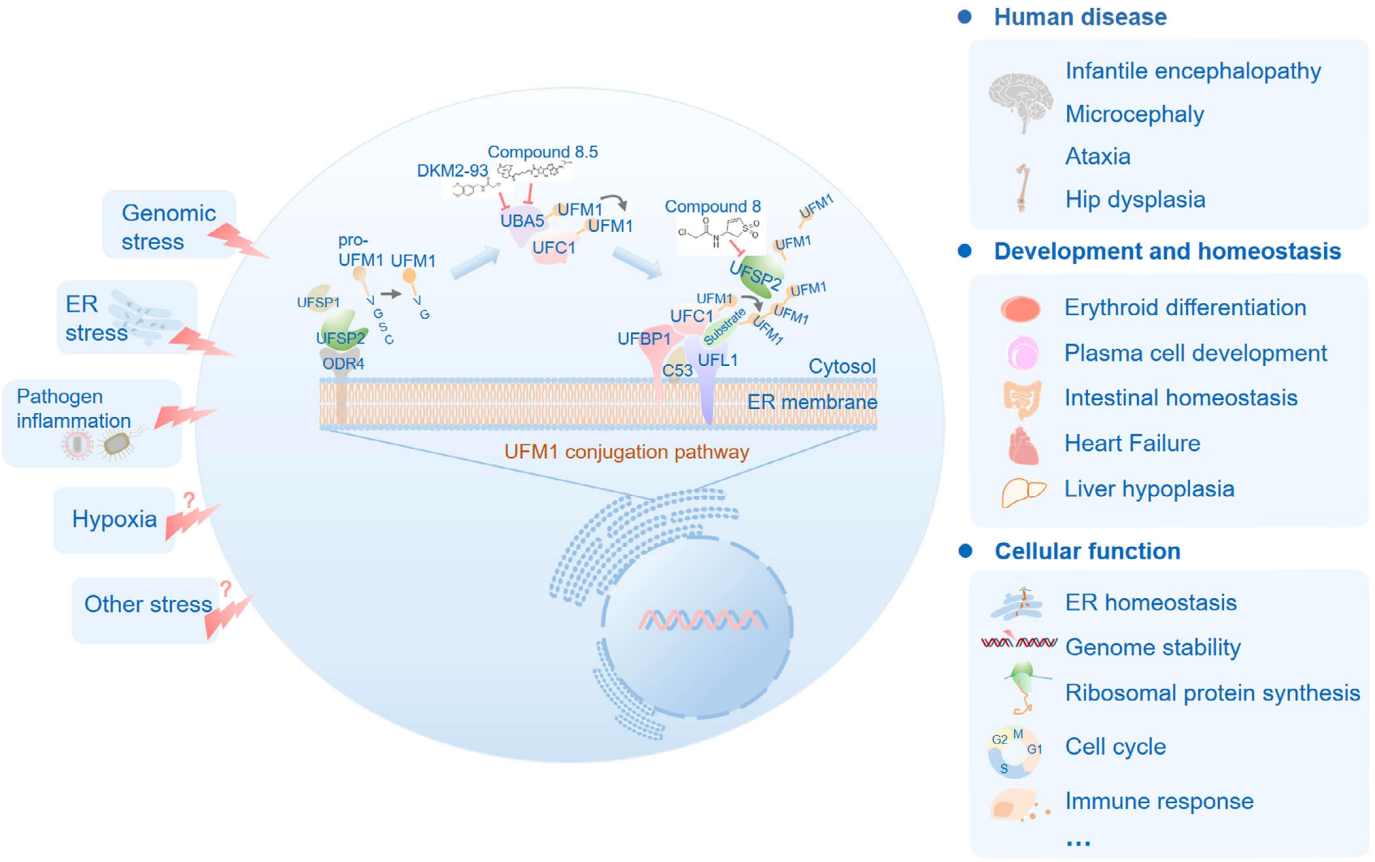
The regulatory system of UFMylation. Pro‐UFM1 is cleaved by UFSP1 and UFSP2 in its C‐terminal region, which generates the mature UFM1 by exposing the C terminus glycine residue. UBA5 activates UFM1, and transfers to UFC1, the E2 conjugating enzyme. Subsequently, the UFM1‐UFC1 thioester intermediate is transferred to a stable UFM1‐specific E3 enzyme complex comprising UFL1 and UFBP1. This complex is anchored on the cytosolic side of the ER. CDK5RAP3 (C53 in the Figure) acts as a stabiliser for the complex at the ER. Both UFBP1 and CDK5RAP3 are possible adaptor proteins that allow the ligase UFL1 to recruit a wider pool of substrates. Eventually, the substrates become mono‐ or poly‐UFMylated. The UFMylated substrates are cleaved mainly by UFSP2, which is a major de‐UFMylation enzyme of the pathway, and forms a complex with ODR4 to localise to the ER. Consistent with conservation of the UFM1 system, genetic screenings across diverse human populations have identified variants of the UFM1 system genes in neurodevelopmental diseases, including infantile encephalopathy, microcephaly and ataxia,^39^, ^40^, ^41^ hip dysplasia^42^, ^43^ and carcinomas.^104^ Genetic deletion of key UFM1 system components, such as UBA5, UFL1, UFBP1 or CDK5RAP3, revealed that the UFM1 system is critical to mice embryonic development, plasma cell development, haematopoiesis, erythroid differentiation, liver development, intestinal homeostasis, heart failure and zebrafish epiboly.^12^, ^16^, ^29^, ^31^, ^44^, ^45^, ^46^, ^47^ Importantly, the identification of numerous UFMylation substrates underscores the critical role of UFMylation in multiple cellular functions, such as ER homeostasis, including ER‐RQC and ER‐phagy, genomic stability, tumorigenesis and immune response.

UFMylation involves a three‐enzyme cascade (E1, E2 and E3), with only one enzyme of each discovered so far (Figure 1). UFM1 is initially synthesised as a precursor molecule, proUFM1, which then undergoes processing by UFM1‐specific protease 1 (UFSP1) and UFM1‐specific protease 2 (UFSP2),^20^, ^21^, ^22^, ^23^, ^24^ resulting in a mature UFM1 with a unique sequence of Val‐Gly.^4^, ^22^ Following maturation, the ubiquitin‐activating enzyme 5 (UBA5), E1 of the pathway, activates the C‐terminus of UFM1.^4^ Then, UFM1 is transferred onto the E2 UFM1‐conjugating 1 (UFC1).^25^ While the majority of the UFM1 system is predominantly located at the cytosolic side of the endoplasmic reticulum (ER) membrane, UBA5 and UFC1 are distributed in the cytosol and nucleus.^4^ In the final step, E3 UFM1 ligase 1 (UFL1), acting as a scaffold‐like E3, encourages the substrate and E2 to come into close contact, and enables the transfer of UFM1 from E2 to lysine residues on substrate proteins.^26^ It has been demonstrated that UFSP2 is the main ‘deUFMylation’ enzyme compared with the inactive UFSP1.^4^ However, recent studies demonstrated that human UFSP1 can reverse ASC1 UFMylation and is required to remove a possible self‐inhibitory modification on UFC1, indicating that UFSP1 act at different points to secure proper UFMylation.^21^, ^23^ Further research is required to determine whether human UFSP1 is actually catalytically active across different cell types or under diverse cellular conditions.

The UFM1‐binding protein 1 (UFBP1, also known as DDRGK1 or C20orfl16, was recognised as the first UFMylation substrate, whose UFMylation is necessary for several substrates, such as ASC1 and RPN1.^27^, ^28^ Besides, UFBP1 emerged as an interactor and adaptor of UFL1.^26^, ^29^, ^30^ In the absence of UFBP1, UFL1 is unstable and lacks activity; however, the presence of UFBP1 gives rise to a stable complex that has the ability to facilitate substrate UFMylation.^30^ Thus, UFBP1 may play a dual role in UFMylation, functioning both as a substrate itself and as a cofactor that facilitates the other substrates’ UFMylation. The second identified adaptor protein for UFL1 is the CDK5 regulatory subunit‐associated protein 3 (CDK5RAP3, also known as C53 or LZAP).^31^ CDK5RAP3 interacts with UFL1, thereby stabilising itself and facilitating its relocation to the ER in conjugation with the E3 complex.^32^, ^33^ Depletion of CDK5RAP3 results in a decrease of di‐UFMylated RPL26, indicating that CDK5RAP3 could be a necessary component for the assemble of poly‐UFMylation chains.^32^ Additionally, CDK5RAP3 was initially described as a tumour suppressor,^14^, ^34^ while subsequent research also revealed its role in promoting tumorigenesis.^35^, ^36^ The role of CDK5RAP3 in human cancers is still being debated. Deeper exploration is necessary to unravel the exact molecular mechanisms that govern its diverse functions. Last but not least, studies have highlighted the critical role of the odorant response abnormal protein 4 (ODR4) in the proper localisation of UFSP2 to the ER.^23^, ^37^, ^38^ Both UFSP2 and ODR4 contain an Mpr1p and PAD1p N‐terminal (MPN) domain, allowing them to form a dimeric complex.^37^ Further experimental evidence confirmed the direct association of UFSP2 and ODR4, which is essential for the ER localisation of UFSP2, given its lack of a transmembrane domain.^23^, ^37^ The interaction between ODR4 and UFSP2 also appears to provide mutual stabilisation, as the expression of ODR4 is significantly lower in UFSP2‐deficient cells, and UFSP2 expression levels are also reduced in cells lacking ODR4.^23^ It is noteworthy, however, that these investigations have solely focused on UFSP2 in isolation, rather than in conjugation with ODR4. Further research is necessary to establish if ODR4 also influences the activity of UFSP2, in addition to its known role in localisation and stability.

The conservation of the UFM1 system highlights its essential and unique role in human development and disease. Consistent with this hypothesis, genetic screenings across diverse human populations have revealed variants of the UFM1 system genes in neurodevelopmental disorders, such as microcephaly, infantile encephalopathy and ataxia (e.g., UBA5^c.1111G>A^, UBA5^c.760A>G^),^39^, ^40^, ^41^ and hip dysplasia (e.g., UFSP2^c.1277A>C^, DDRGK1^c.408+1G>A^).^42^, ^43^ Genetic deletion of key UFM1 system components, such as UBA5, UFL1, UFBP1 or CDK5RAP3, leads to embryonic lethality,^29^, ^31^, ^44^, ^45^, ^46^ heart failure^47^ and imbalance of intestinal homeostasis.^16^ Importantly, the identification of numerous UFMylation substrates underscores its critical role in multiple cellular functions (Table 1, Figure 1). For instance, the UFM1 system contributed to the processes in maintaining ER homeostasis, including ER‐ribosome‐associated quality control (ER‐RQC)^32^, ^48^, ^49^, ^50^, ^51^, ^52^, ^53^ and ER‐phagy.^28^, ^54^, ^55^, ^56^ By targeting nucleus localised substrates, UFMylation plays a pivotal role in maintaining genomic stability.^57^, ^58^, ^59^, ^60^, ^61^, ^62^ Meanwhile, UFMylation has emerged as a crucial factor in cancer development.^10^, ^27^, ^63^, ^64^

**TABLE 1:** Key substrates or binding proteins of the UFM1 system.

Substrates	UFMylation sites	Involved biological processes	UFMylation functions
STING	Independent on UFMylation	HSV‐1 infection in macrophages	UFL1 stabilises STING by preventing its interaction with the E3 ubiquitin‐protein ligase TRIM29 to activate antiviral immunity
MAVS	K461	EBV replication in Burkitt lymphoma cell lines	BILF1 mediates MAVS UFMylation‐dependent immune evasion
PDL1	K75,89,105, 162, 280, 281	Breast cancer	Inhibition of PD‐L1 UFMylation stabilises PD‐L1 and undermines anti‐tumour immunity
PD1	K210,233	Colon cancer	UFMylation of PD1 inhibits PD‐1 ubiquitination and degradation and promotes T‐cell anti‐tumour activity
UFBP1	K267	B‐cell development and function	UFMylation of UFBP1 is necessary for the production of immunoglobulin and the expansion of ER in IRE1α‐deficient plasmablasts
14‐3‐3ε	Not available	SeV infection in 293T cells	14‐3‐3ε UFMylation is required for the interaction with activated RIG‐Ι to promote antiviral immunity
RPL26	K134,132, 134, 136, 142	ER proteostasis	RPL26 UFMylation controls protein synthesis, ER protein translocation, RQC and ER‐phagy
RPN1	Not available	ER‐phagy	UFMylation of RPN1 upon ribosome collision is involved in ER‐phagy with unknown mechanisms
CYB5R3	K214	ER‐phagy	CYB5R3 can be UFMylated to induce ER‐phagy, which is essential for brain development
H4	K31	DNA damage repair	H4 UFMylation is responsible for the activation of ATM pathway and maintenance of genomic stability
MRE11	K281, 282	DNA damage repair	MRE11 UFMylation is necessary for the assembly of MRN complex and activation of ATM
PTIP	K148	Replication stress	PTIP UFMylation promotes the degradation of replication fork in BRCA1‐deficient cells
PARP1	K548	Replication stress	UFMylation of PARP1 can enhance its catalytic activity during replication stress
P53	K351, 357, 370, 373	Colon cancer	UFMylation of tumour suppressor p53 can stabilise p53 by countering its ubiquitination and proteasome degradation
PLAC8	K103	TNBC	UFMylation of PLAC8 helps maintain its stability and upregulate PD‐L1 expression to promote TNBC cell proliferation
RPL10	Not available	Pancreatic cancer	UFMylation of RPL10 facilitates the proliferation and stemness in pancreatic cancer cells
ERα	K171, 180	Breast cancer	UFMylation of ERα can promote its stability and transactivity by inhibiting its ubiquitination and degradation, and facilitating breast cancer progression
ASC1	K324, 325, 334, 367	Breast cancer	ASC1 UFMylation can promote transcription factors binding to ERα and facilitates breast cancer development
SLC7A11	Not available	Ferroptosis	The anticancer effect of metformin was accomplished by the inhibition of SLC7A11 UFMylation
P4HB	K69, 114, 130	ER stress, mitochondrial function, and oxidative stress	UFMylation of P4HB helps maintain its stability and biological function in HepG2 cells

First and foremost, ribosomal protein L26 (RPL26/uL24) is one of the principal cellular substrates of the UFM1 system.^32^, ^48^, ^49^, ^50^, ^51^ UFMylated RPL26 contributes to a protein quality control system that ensure the proper co‐translational translocation of proteins into the ER, a critical step in protein biogenesis. In the progression of protein translocation, ribosome stalling triggers RPL26 UFMylation. Deletion of UFL1 or disrupting its interaction with UFBP1 results in the loss of RPL26 UFMylation, while in cells lacking UFSP2, there is a significant enhancement in the sequential UFMylation of the two specific lysine residues, K132 and K134, on RPL26.^32^ Sequential cryo‐electron microscopy snapshots showed that after catalysing UFM1 transfer, UFL1 remained stably interactive with its product, formed a C‐shaped clamp that wraps around the 60S from the transfer RNA‐binding sites to the polypeptide tunnel exit, and promoted recycling of RPL26 from the ER.^48^ In addition, the UFMylation sites on RPL26 are located near the interface between the exist tunnel of nascent peptides and the SEC61 translocon.^32^ Recent analyses demonstrated that the UFMylation of RPL26 is indispensable for releasing SEC61 from 60S subunits.^49^ Meanwhile, RPL26 UFMylation promoted the translocation‐arrested peptides transported to lysosomes for degradation mediated by SAYSD1.^51^, ^52^ Apart from RPL26 UFMylation mediated ER‐ribosome‐associated quality control (ER‐RQC) system, the control of ER proteostasis by UFMylation is achieved through several other mechanisms. UFBP1 is the first identified substrate of UFMylation.^26^ Through regulating different branches of unfolded protein response (UPR) pathway in plasmablasts lacking IRE1α, UFBP1 UFMylation is not necessary for the plasmablast development, but is essential for ER expansion and immunoglobulin synthesis.^12^ Prolyl 4‐hydroxylase beta (P4HB), present in the ER, mitochondria and cytosol, can be mono‐UFMylated at K69, K114 and K130.^65^ Defective UFMylation of P4HB causes ER stress in HepG2 cells. The connection between the UFM1 system and ER‐phagy was first uncovered with the discovery of the UFM1 E3 complex in a genome‐wide screen of factors crucial for ER‐phagy.^28^ The UFMylation of RPL26, CYB5R3 and a component of the oligosaccharyl‐transferase (OST) complex, RPN1, is considered to contribute to ER‐phagy, though the exact mechanisms are still unclear.^28^, ^54^

In addition to maintaining ER homeostasis, emerging evidence implies that UFMylation controls genome stability. The K31 residue on histone H4 can be mono‐UFMylated by UFL1 upon DNA damage.^58^ This modification is critical for enhancing the activation of ataxia‐telangiectasia mutated (ATM) kinase, responsible for the orchestration of DNA repair and the maintenance of genomic stability. The discovery that serine/threonine kinase 38 (STK38) function as a reader for UFMylation on histone H4 suggests the potential necessity for a broad range of readers, writers or erasers to recognise the attached UFM1 on targets and manage their biological roles.^66^ Meiotic recombination 11 homolog 1 (MRE11) undergoes UFMylation on residue K282 in response to DNA double‐strand breaks (DSBs).^60^ This modification is essential for the assembly of MRE11‐RAD50‐NBS1 (MRN) complex and its subsequent recruitment to the site of DNA damage.^60^ Defective MRE11 UFMylation can disrupt the activation of ATM and compromise the genome stability.^59^, ^60^ The tumour suppressor p53 is also a target for covalent modification by UFM1 at K351, K357, K370 and K373 upon DNA damage, leading to the stabilisation of p53 by the inhibition of its ubiquitination and proteasome degradation.^57^ The stability of the replication fork is another critical aspect of genome integrity. Poly[ADP‐ribose] polymerase 1 (PARP1), a sensor of replication stress, can be UFMylated at K548, enhancing its catalytic activity during replication stress.^61^ Defective PARP1 UFMylation can lead to excessive degradation of nascent DNA at stalled replication forks.^61^ Moreover, in the context of resistance to PARP inhibitors, the loss of UFL1, disruption of pax2 transactivation domain interacting protein (PTIP) UFMylation, or UFSP2 overexpression is capable to prevent nascent DNA strands from excessive degradation, and impart the resistance in cells with BRCA1/2 deficiency.^62^, ^67^ These studies underscore the importance of UFMylation in maintaining genomic stability. Although these studies provide compelling evidence for nuclear roles of UFMylation, the mechanism by which nuclear UFMylation is catalysed remains elusive, particularly considering that UFBP1 is anchored on the cytosolic side of ER membrane, which is responsible for maintaining the stability and activity of UFL1.

To date, a multitude of aberrant UFM1 modifications have been discovered in tumorigenesis. The estrogen receptor α (ERα), one of the substrates of UFMylation in breast cancer, experiences reduced stability upon UBA5 deletion, but is enhanced by UFSP2 depletion.^68^ The activating signal co‐integrator 1 (ASC1), a transcriptional coactivator for Erα, has been demonstrated to be modified by the UFM1 at K324, K325, K334 and K367.^27^ Poly‐UFMylated ASC1 promotes the assembly of p300, SRC1, as well as itself at the promoters of ERα target genes, thereby influencing tumour formation. The UFMylation of solute carrier family 7 member 11 (SLC7A11), the cysteine transporter, has a crucial impact on breast cancer tumorigenesis.^69^ By inhibiting SLC7A11 UFMylation, metformin exerts its anticancer effects. Placenta associated 8 (PLAC8), a recently identified substrate of UFMylation, has been implicated in breast cancer pathogenesis.^63^ UFMylation of PLAC8 at K103 helps maintain its stability and upregulates PD‐L1 expression, which promotes the proliferation of triple‐negative breast cancer (TNBC) cells, and suppresses the activity of T cells.^63^ Additionally, in pancreatic cancer, UFMylation of ribosomal protein RPL10 has been found to significantly facilitate the proliferation and stemness of tumour cells.^70^

Recent studies are shedding new light on the emerging roles of UFMylation in regulating immune response, including antiviral immune defence,^7^, ^8^, ^9^ PD‐1/PD‐L1 checkpoint modulation,^10^, ^11^ B‐cell development and function,^12^ and inflammatory responses,^13^, ^14^, ^15^ suggesting another potential mechanism that could account for the indispensable role of UFMylation in maintaining tissue homeostasis. In this review, we concentrate on the newly uncovered principles of the UFM1 system's involvement in both innate and adaptive immunity, the immune disorders directly linked to defective UFMylation, and the potential roles in the crosstalk between the endocrine system and immune responses.

## 3 UFMYLATION IN THE CONTROL OF INNATE IMMUNITY AND INFLAMMATORY RESPONSES

**FIGURE 2:**
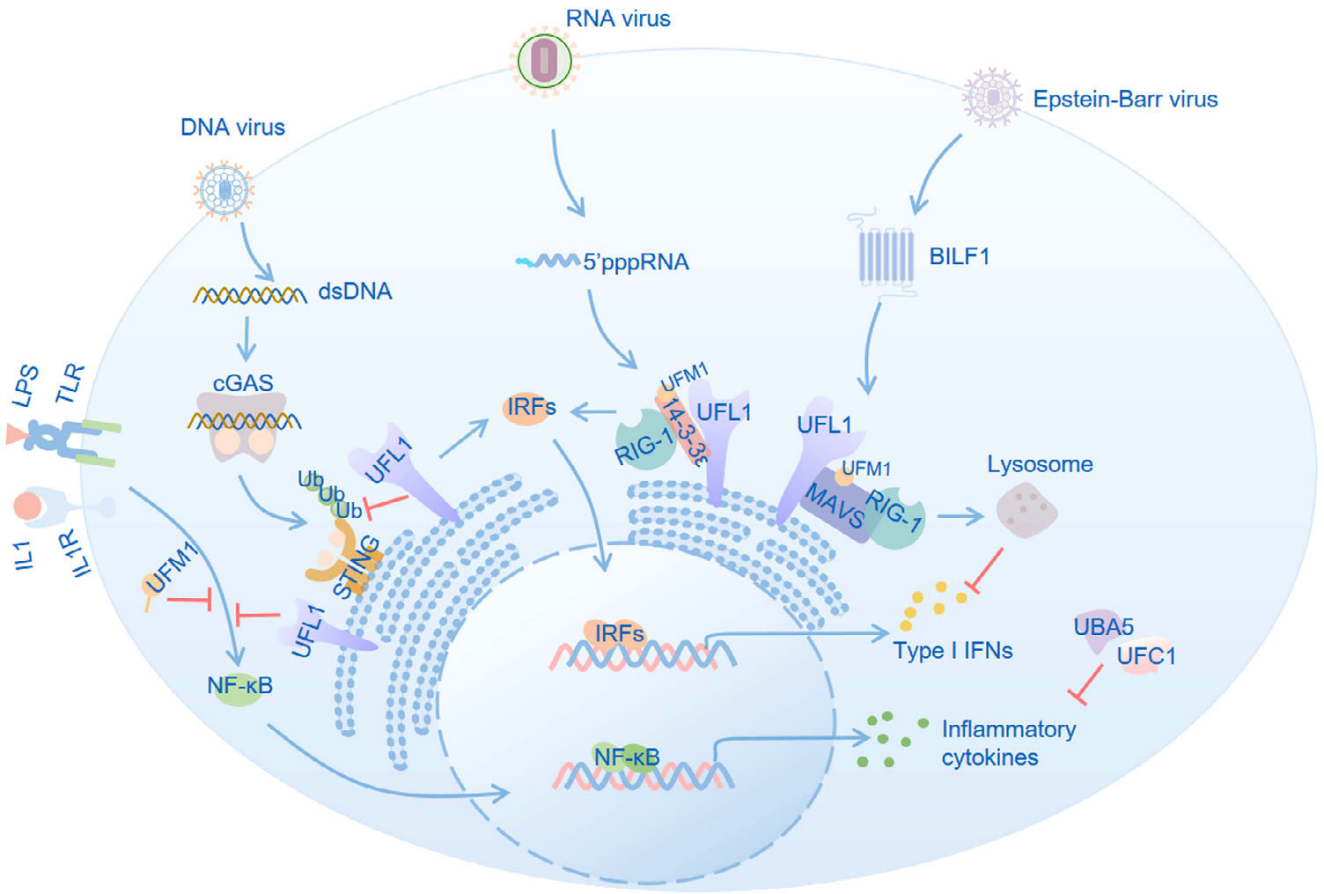
Functional regulation of UFMylation in innate immunity. The UFM1 system regulates antiviral responses and inflammatory pathways. UFL1 interacts with STING to prevent its ubiquitination and proteasomal degradation upon HSV‐1 infection.^9^ Increased interaction between 14‐3‐3ε and UFL1 leads to UFMylation and subsequent binding to K63‐ubiquitinated RIG‐Ι, enhancing downstream IFN signalling during SenV infection.^7^ The EBV‐encoded BILF1 mediates MAVS UFMylation, targeting it for lysosomal degradation and potentially disrupting NLRP3 inflammasome activation and IRF3 responses.^8^ The UFM1 system suppress the proinflammatory capacity of IFN‐γ or LPS‐mediated macrophage activation,^13^ and inhibits NF‐κB activation with unclear mechanisms.^77^, ^79^ During AS development, UFM1 plays a role in reducing foamy cells formation by activating LXRα and supressing the expression of pro‐inflammatory cytokines produced by LPS‐activated endothelial cells.^18^, ^77^ In mucosal barrier, UFBP1 deletion results in ER‐stress‐induced apoptosis in Goblet and Paneth cells, alterations in the faecal microbiota and increased susceptibility to inflammatory colitis.^16^

The host's innate immune system provides a critical barrier of defence. When pathogens overcome physical barriers, innate immune cells detect ‘non‐self’ infectious and danger signals through pattern recognition receptors (PRRs), such as Toll‐like receptors (TLRs), retinoic‐acid‐inducible gene I (RIG‐I)‐like receptors (RLRs), nucleotide‐binding domain and leucine‐rich repeat containing molecules (NLRs) and cytoplasmic DNA sensors. Upon ligand binding, PRRs initiate diverse and interactive signalling networks that typically culminate in the expression of inflammatory cytokines or chemokines dependent on the nuclear factor‐κB (NF‐κB), as well as the expression of type I interferons (IFNs) dependent on the interferon regulatory factors (IRFs). The activated immune signalling results in the engulfment of pathogens, the attraction of immune cells to infection site and the initiation of inflammatory responses. In this section, we will explore the regulation of UFMylation in innate immunity, focusing on three key aspects: antiviral responses, inflammatory pathways and mucosal immunity (Figure 2).

### 3.1 UFMylation regulates IFN‐γ‐mediated antiviral defences

IFNs are critical mediators of cell‐intrinsic immunity against viral challenges.^71^ In the event of an infection, RIG‐I is capable of detecting viral RNA, and the cyclin GMP‐AMP synthase (cGAS) acts as a sensor for cytoplasmic DNA. Typically, the activation of RIG‐1 and cGAS triggers downstream IFN‐mediated antiviral responses, typically through mitochondrial antiviral signalling protein (MAVS) and stimulator of interferon genes (STING) separately.^72^, ^73^

Notably, UFMylation has been recognised as an essential regulator in RIG‐I activation.^7^ Following SenV infection, the interaction between 14‐3‐3ε and UFL1 increases, UFL1 catalyses the UFMylation of 14‐3‐3ε subsequently. The UFMylated 14‐3‐3ε subsequently associates with K63‐ubiquitinated, activated RIG‐Ι to activate the production of IFN‐γ. Conversely, UFMylation deficiency impedes the association of 14‐3‐3ε with RIG‐Ι, ultimately leading to a decrease in IFN‐γ production. Nevertheless, viruses have evolved strategies to evade the antiviral responses. It has been reported that the Epstein–Barr virus‐encoded G protein‐coupled receptor BILF1 was recognised to have an association with MAVS and UFL1 in Burkitt lymphoma cell lines. BILF1 expression is adequate to trigger the dislocation of MAVS from mitochondrial via K461 UFMylation, leading to the encapsulation of MAVS into vesicles derived from mitochondria, and subsequent lysosomal pathway for protein degradation.^8^ There is possibility that the MAVS is incorporated into vesicles and then degraded by the initiation of translocation‐associated quality control (TAQC) on mitochondria via redirecting of UFL1 from the ER to the mitochondria mediated by BILF1, although further investigation is still required. Interestingly, recent investigations have revealed that UFL1 plays a protective role during DNA viruses’ infection by suppressing the ubiquitination and degradation of STING.^9^ UFL1 deficiency effectively suppresses the activation of IRF3 and NF‐κB signalling pathways in macrophages upon HSV‐1 infection. Further analysis has demonstrated that UFL1 achieves this by competitively binding to STING independent of UFMylation, preventing the interaction between STING and TRIM29, a E3 ubiquitin‐protein ligase, thereby suppressing the ubiquitination and proteasomal degradation of STING.

The aforementioned studies have not only advanced our understanding of the UFM1 system in antiviral immunity, but also illuminated potential approaches to eliminate or restrict viruses that cause serious illness or mortality in human populations. While the majority of research has been conducted on macrophages,^9^, ^13^, ^74^, ^75^ it is now evident that IFN‐γ is produced by various lymphocytes, and hundreds of human cell types expressing the signalling machinery may have the ability to respond to IFN‐γ.^71^ Future work is needed to explore the UFMylation regulatory mechanisms in IFN‐γ response and production across different cell types. It is noteworthy that UFMylation of RPL26 has been shown to enhance the translation of hepatitis A virus in human hepatocytes.^76^ The precise role of UFMylation in the complex interplay between the host's antiviral defences and viral translation in infected cells, and the potential involvement of UFMylation of other substrates associated with ER‐RQC or ER‐phagy, remain to be elucidated.

### 3.2 UFMylation functions in the TLRs and NF‐κB key pathways of inflammation

Inflammatory responses serve as a cornerstone of pathology. It has been revealed that UFMylation acts as a suppressor of the inflammatory responses triggered by IFN‐γ and lipopolysaccharide (LPS).^13^ The enzymatic activities of UFSP2, UBA5 and UFC1 are required in the negative regulation of macrophage activation induced by IFN‐γ and LPS, given that the expression of their dysfunctional mutants did not restrict NO production. Transcriptional profiling in cells with deficient UFMylation has revealed the ER stress response mediated by ERN1, which accounts for the increased sensitivity to pro‐inflammatory stimuli.

Inflammatory responses are typically initiated through TLRs/NF‐κB signalling pathway. Research has established that inflammatory responses induced by LPS can be inhibited by UFM1 in endothelial cells via the NF‐κB signalling pathway.^77^ Overexpression of UFM1 in goat endometrial epithelial cells (gEECs) has been shown to prevent the activation of TLR4 pathway induced by LPS.^78^ Additionally, in chondrocytes, UFL1 suppresses the activation of NF‐κB signalling pathway induced by IL‐1β.^79^ UFL1 also exerts a suppressive effect on the activation of NLRP3 inflammasome, partly through the modulation of NF‐κB signalling.^15^ However, the precise molecular mechanisms by which the UFM1 system modulates the activation of TLRs/NF‐κB is still in its early stages. Some studies have shown that the UFM1 system components can interact with NF‐κB components. For example, LZAP has been demonstrated to bind directly to RelA, thereby inhibiting NF‐κB transcriptional activity.^14^ Similarly, UFM1, along with DDRGK1, has been found to interact with IκBα, resulting in the activation of NF‐κB transcriptional activity.^17^, ^80^ Moreover, UFMylation is capable of modulating inflammation through other mechanisms. UFM1 can impede oxLDL‐induced foam cell formation, decrease the production of inflammatory cytokines and subsequently suppress atherosclerosis (AS) development via liver X receptor α (LXRα)‐dependent pathway.^18^

The intricate interplay between the inflammatory pathway and the UFM1 system offers potential therapeutic targets for managing excessive inflammation and preventing unnecessary cell death. Future investigations are necessary to delineate the precise molecular mechanisms by which the UFM1 system modulates TLRs/NF‐κB, identify key targets within inflammatory pathways that are susceptible to UFMylation, and understand how alterations in UFMylation occur across various pathological contexts. Achieving these will not only broaden our understanding of the fundamental biology of inflammation but also reveal novel therapeutic targets for a wide range of inflammatory diseases.

### 3.3 UFMylation participates in the biological functions of mucosal barrier

Intestinal mucosal immunity represents the largest peripheral lymphoid organ in animals and serves as a critical site for the body's immune defence.^81^ Specialised intestinal epithelial cells, such as Paneth cells, are capable of secreting a variety of immune mediators that help regulate host immune responses. Knockdown of UFBP1 results in a reduction in Paneth cells through the acceleration of ER‐stress‐induced apoptosis.^16^ Furthermore, deletion of UFBP1 caused alterations in the faecal microbiota and increased the susceptibility to inflammatory colitis, highlighting a direct correlation between UFMylation and mucosal immunity, which is crucial for developing strategies to treat immune disorder diseases that affect mucosal tissues, such as inflammatory bowel disease.

## 4 UFMYLATION EXERTS MULTIFARIOUS FUNCTIONS IN THE ADAPTIVE IMMUNITY

**FIGURE 3:**
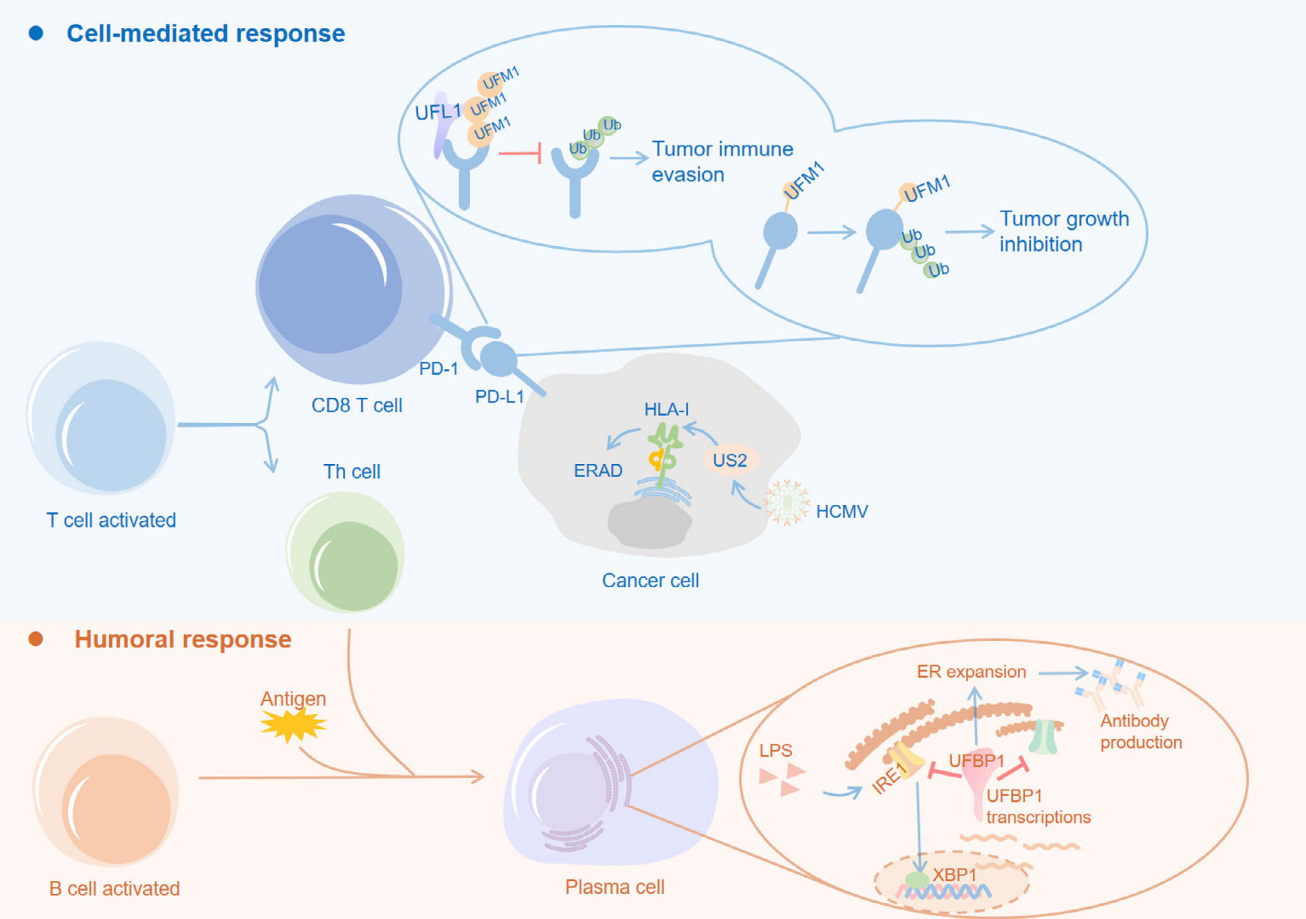
Functional regulation of UFMylation in adaptive immunity. UFMylation has emerged as a key regulator in tumour progression through modulating PD‐1/PD‐L1 signalling pathway. UFMylation tags PD‐L1 for proteasome‐mediated degradation to inhibit tumour growth.^10^ UFL1 interacts with PD‐1 to promote its UFMylation, stabilising PD‐1 and impairing CD8^+^ T‐cell activation.^11^ B‐cells stand as pivotal players in the generation of specific antibodies. The differentiation of plasma B cells stimulates a transcriptional increase of UFBP1 through XBP1s pathway. Serving as a feedback inhibitor, UFBP1 constrains the activity of sensors IRE1 and PERK, fostering ER expansion and immunoglobulin production.^12^ Classical HLA‐I molecules bind with antigens and present them to T cells. The UFM1 system accelerates ERAD of HLA‐I with unraveling mechanisms, facilitating HCMV evasion from immune surveillance.^88^

The complete elimination of pathogens is primarily carried out by the adaptive immune system, mainly driven by T cells and B cells.^82^ The anti‐pathogen or anti‐tumour reactivity of T cells is defined by their unique T‐cell receptors (TCRs), which can recognise specific antigens presented by human leukocyte antigen (HLA) complex.^82^ The antibody‐mediated humoral immune response of B cells is characterised by their unique B‐cell receptors (BCRs), enabling them to identify specific antigens and facilitate the production of highly specific antibodies.^82^ The UBL modifications are integral to regulate the biology of T cells and B cells.^83^, ^84^ In light of this aspect, we aim to present an overview of how the UFM1 system impacts adaptive immunity, including the development and function of B cells, PD‐1/PD‐L1 checkpoint‐mediated anti‐tumour immune responses, and the HLA‐I‐mediated antigen presentation pathway (Figure 3).

### 4.1 UFMylation core component UFBP1 regulates the function of B cells

B cells are essential to the immune system, fulfilling vital roles within the adaptive immune response. Upon activation, B cells differentiate into plasma cells that produce and secret large amounts of antibodies into the bloodstream to recognise and neutralise specific antigens, as well as into memory B cells that ensure long‐term immunity.^82^ It has been demonstrated that UFBP1 distinctly promotes the development and function of plasma cells by modulating different branches of the UPR pathways.^12^ A conditional knockout of UFBP1 in vivo resulted in significantly reduced levels of serum immunoglobulins, specific antibodies and the conversion of activated B cells into plasma cells following immunisation, despite maintaining a normal number of B cells, indicating that the expression of UFBP1 in B cells is required for the production of serum immunoglobulins and the initiation of antibody response. To provide further illumination on the regulation of plasma cell development and function mediated by UFBP1, the ER network in UFBP1‐deficient plasmablast was investigated. UFBP1 induces PERK suppression during plasma cell differentiation, a process independent of UFBP1 UFMylation at K267. Furthermore, the absence of UFBP1 inhibits ER expansion and immunoglobulin production in plasma cells. On the other hand, the IRE1α/XBP1 pathway was shown to stimulate the expression of UFBP1 and UFMylation system genes. This research sheds light on the underlying molecular and cellular factors that regulate the differentiation and function of plasma cell, offering valuable insights for the design of vaccines that elicit robust humoral responses and for the development of approaches targeting harmful plasma cells.

### 4.2 UFMylation regulates the tumour immune evasion via the PD‐1/PD‐L1 checkpoint

Under physiological conditions, T‐cell stimulation triggered by antigens leads to immune responses mediated by effector T lymphocytes and memory T cells.^82^ PD‐L1, an important immune checkpoint molecule, is frequently overexpressed in various tumours and delivers inhibitory signals to T cells via PD‐1 receptor.^85^ Over the past decade, immunotherapy targeting PD‐1/PD‐L1 checkpoint has seen remarkable breakthroughs, contributing to the improved clinical outcomes and prolonged survival for many cancer patients.^85^ However, the response rate to PD‐1/PD‐L1 blockade immunotherapy is less than 15% for most cancer types,^85^ suggesting that multiple factors impact the host's anti‐tumour immune responses. In order to enhance the clinical benefits of PD‐1/PD‐L1 blockade immunotherapy, further in‐depth investigation of the mechanisms underlying anti‐tumour immunity, clinical response and resistance to the therapy is therefore urgently needed.

Our previous study identified PD‐L1 as a substrate for UFMylation.^10^ The specific mutations of five lysine residues (K75/89/105/162/280/281) on PD‐L1 markedly reduced its UFMylation, indicating that these lysine residues are the sites of UFMylation for PD‐L1. To elucidate the biological function, we demonstrated that the disruption of UFMylation inhibits the ubiquitination of PD‐L1, thus preserving its stability by preventing proteasome‐mediated degradation. UFSP2 is necessary for the removal of UFM1 from the UFMylated PD‐L1. Clinically, compound‐8, a covalent inhibitor of UFSP2, was found to promote PD‐L1 UFMylation significantly without influencing UFSP2 expression. Furthermore, compound‐8 contributes to combination treatment with PD‐1 blockade in vivo, revealing a novel modulator of PD‐L1 and highlighting UFMylation as a possible immunotherapy target. In addition, a previous study showed that UFM1 could upregulate PD‐L1 expression by stabilising PLAC8,^63^ indirectly supporting the notion that targeting the UFM1 system may enhance the efficacy of current immune checkpoint blockade therapy and boost anti‐tumour responses. A recent study has investigated UFM1 modification within T cells, adding another layer to our understanding of the UFM1 system's role in PD‐1/PD‐L1 checkpoint modulation.^11^ Using a proteomic interaction approach, the study demonstrated that UFL1 can interact with PD‐1 to promote its ubiquitination and subsequent degradation, typically downregulating the immune‐inhibitory signal mediated by PD‐1 and potentially facilitating T‐cell activity against cancer cells. A conditional knockout of UFL1 in vivo led to slower tumour growth in comparison with wild‐type mice, suggesting that the deletion of UFL1 increases T‐cell activity within the tumour, and leads to a more robust anti‐tumour response. Single‐cell sequencing data indicated that absence of UFL1 in T cells increased the infiltration of cytotoxic CD8^+^ T cells, consistent with the observed enhanced anti‐tumour effect. Furthermore, the study proposes that AMPK could phosphorylate UFL1 at Thr536, disrupting the UFL1‐PD1 interaction and increasing the stability and PD‐1 expression. Modulation of the AMPK‐UFL1‐PD‐1 axis directly impacts anti‐tumour immune responses. Combining an AMPK agonist with CTLA‐4 antibody treatment showed a synergistic effect in controlling tumour development. In summary, the study highlights a novel regulatory mechanism of T‐cell‐mediated immunity and its potential for exploitation in cancer therapy.

Overall, targeted therapy against the UFM1 system could be a complementary approach to existing PD‐1/PD‐L1‐based tumour immunotherapy. The modulation of the UFM1 system presents an innovative therapeutic strategy, which could help reduce PD‐1/PD‐L1 expression and, consequently, enhance the ability of the immune system to target and kill tumour cells.

### 4.3 UFMylation participates in antigen presentation pathway

Classical HLA‐I molecules are capable of binding a broad array of peptides derived from the cytosolic and endosomal degradation of proteins, and presenting them to T cells, which plays a fundamental role in adaptive immunity.^86^ The UFM1 system is an important regulatory mechanism that maintains ER homeostasis.^12^, ^51^, ^87^ Given the essential role of the ER in the antigen presentation machinery (APM),^86^ any disruptions in ER function or homeostasis could potentially affect the APM process. It has been shown that UFMylation accelerates ER‐associated protein degradation (ERAD) of HLA‐I molecules with unknown mechanisms, thereby facilitating human *Cytomegalovirus* (HCMV) evasion from immune surveillance.^88^ Unraveling the regulatory mechanism by which the UFM1 system modulates the efficiency of antigen presentation has the potential to enhance immune surveillance against microorganism infections and even cancer cells.

## 5 UFMYLATION MODIFICATION REGULATES THE ENDOCRINE SYSTEM

**FIGURE 4:**
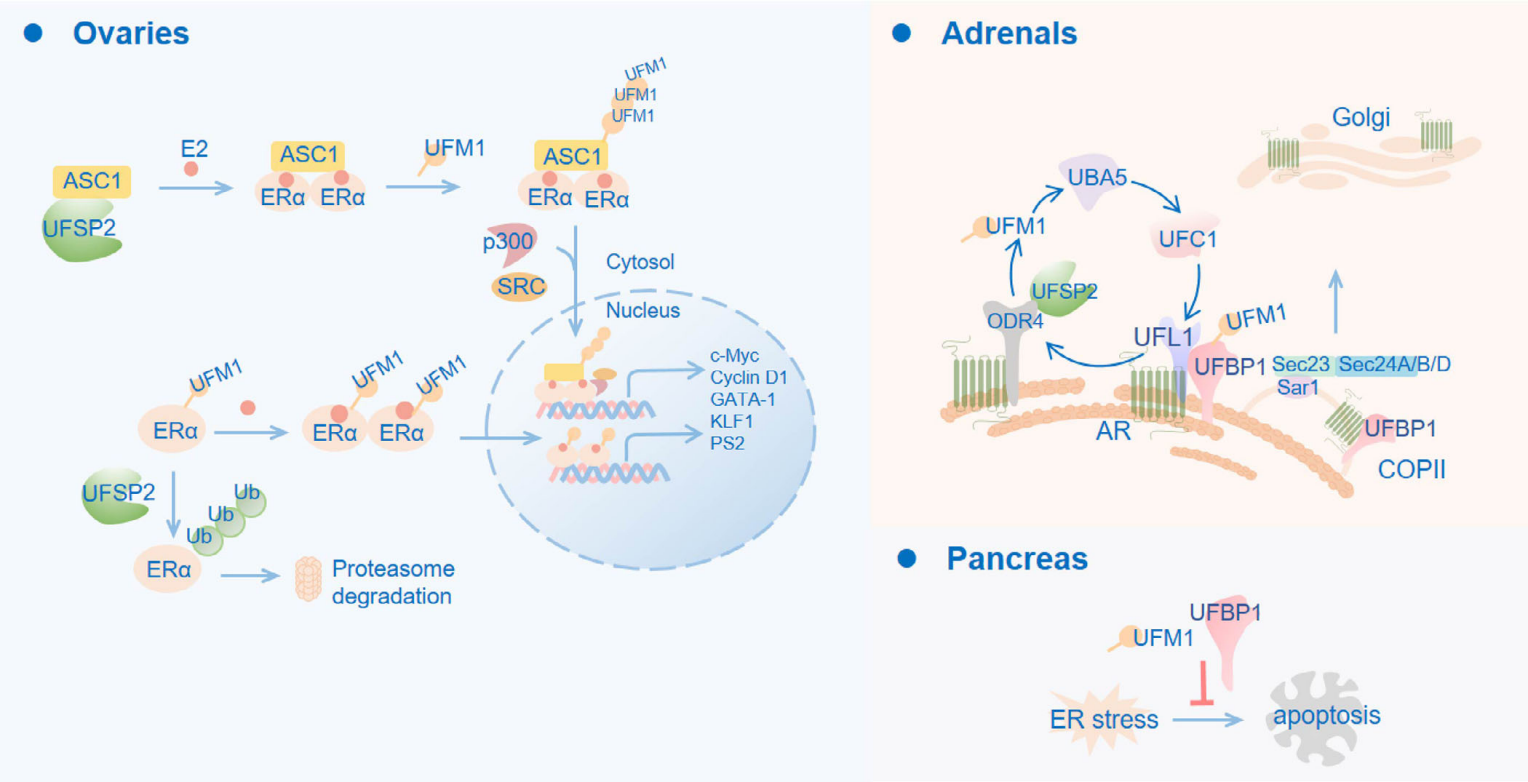
Functions of UFMylation in the endocrine system. The endocrine system crosstalks with the immune system through hormonal networks. UFM1 knockout impedes the ER‐Golgi transport of ARs.^38^ ERα could be UFMylated to promote its stability and transactivity,^68^ and ASC1 UFMylation is necessary for the transactivation of ERα in the presence of E2.^27^ In pancreas, UFMylation components are overexpressed and protect pancreatic β cells from ER stress‐induced apoptosis.^96^

The endocrine system interacts with the immune system through hormonal feedback mechanisms.^89^ Theoretically, the production of hormones by endocrine secretory cells is fundamentally dependent on robust ER function.^90^ As previously mentioned, UFMylation participates in the maintenance of ER homeostasis. In light of this, we overviewed the involvement of UFMylation in hormone production and function, as well as its contribution to endocrine gland homeostasis (Figure 4).

Estrogen, predominantly produced in the ovaries, plays a critical role in biological processes through its interaction with ERs.^89^ Research indicated that UFL1 protects ovaries from LPS‐induced apoptosis and drug‐induced premature failure by regulating ferroptosis and ER stress.^91^, ^92^ The impact of UFL1 deficiency on estrogen levels produced by the ovaries remains unclear. Investigating the influence of UFL1 on ovarian hormone levels is essential for a comprehensive understanding of female reproductive health. In addition, ERs are recognised as key drivers in the development of breast cancers.^89^ Research has demonstrated that UFMylation of ASC1 is a necessary process for the transactivation of ERα in the presence of 17β‐estradiol (E2).^27^ Building on this, subsequent research uncovered the direct UFMylation of ERα at K171 and K180.^68^ These findings suggest a novel regulatory mechanism for ERα in breast cancer development. It is crucial to further explore how the modification of ERα affects tumour immunology, which could uncover new avenues for therapeutic strategies. The adrenal glands are crucial endocrine organs that play critical roles in maintaining homeostasis through the secretion of various hormones. Emerging evidence indicates that UFMylation contributes to adrenal glands function by regulating ER stress pathway.^93^, ^94^ Notably, ODR4 plays an important role in the transport of newly synthesised adrenergic receptors (ARs).^37^ A recent study indicated that UFM1 knockout impedes the ER‐Golgi transport of ARs.^38^ Altogether, these findings point to a possible mechanism by which the UFM1 system participates in AR‐mediated hormonal signalling and immune responses. Lastly, the pancreas, especially pancreatic β cells, is well‐known for its endocrine function, which is necessary in controlling blood glucose levels.^95^ UFMylation components have been shown to protect β cells from apoptosis.^96^ UFM1 and DDRGK1 expression were increased in response to feeding and ER stress in pancreatic β cells. Impaired UFMylation can lead to apoptosis of pancreatic β cells induced by ER stress. However, neither UFM1 nor DDRGK1 is required for insulin release mediated by glucose. In summary, while the functions and mechanisms of the UFM1 system in endocrine‐immune crosstalk remains largely unexplored, it is an emerging field holding the potential to offer new perspectives on endocrine‐immune interaction, illuminating novel therapeutic avenues for treating a variety of endocrine‐immune disorders.

## 6 POTENTIAL VALUES OF UFMYLATION IN CLINICAL DIAGNOSIS AND TREATMENT

Clinical studies have identified that genetic variants within the UFM1 system lead to human pathogenesis, including hip dysplasia and neurodevelopmental disorders.^39^, ^40^, ^41^, ^42^, ^43^ Meanwhile, numerous studies have reported abnormal expression patterns of UFM1 system components in diseases samples, including non‐alcoholic fatty liver disease (NAFLD) and various tumours.^10^, ^68^, ^97^ Moreover, somatic copy number alterations (SCNAs) analysis has uncovered that UFSP2 was heterozygous loss in colon cancer.^98^ These findings suggested that the UFM1 system may serve as a promising genomic biomarker for human diseases.

Therapeutically, a covalent inhibitor of UFSP2, known as compound‐8, has been shown to promote PD‐L1 UFMylation and contribute to combination in vivo treatment with PD‐1 blockade.^10^ Inhibitors targeting UBA5, such as DKM2‐93 and compound 8.5, could influence the subsequent UFMylation steps and impede tumour growth.^99^, ^100^ In parallel, patents have proposed innovative prevention and therapeutic strategies for diseases by engaging the UFM1 system (worldwide.espacenet.com). For example, DDRGK1 has been developed for its potential in predicting cancer prognosis and treating bone or joint diseases in mice (CN111879950A, WO2021098240A1 and CN115975045A). However, to date, neither the aforementioned small molecule inhibitors nor the associated patents have advanced to clinical trials (clinicaltrials.gov).

Based on the protein structure and AI algorithm (e.g., Alphafold),^30^, ^101^, ^102^ we believe that more effective compounds targeting UFMylation will be generated in the future, and further clinical investigation is needed to investigate the viability and efficacy of these potential clinical applications.

### 6.1 Concluding remarks

As a frontier in the study of UBL modification, UFMylation is a brand new and highly attractive research field. Over recent decades, remarkable progress has been made in the clarification of the UFM1 system, establishing its critical role in the function and maintenance of multiple cellular processes. Nonetheless, there are still many more questions remain to be addressed: What are the machinery mechanisms regulating the expression levels and activity of the UFM1 system components? Are there additional proteins that participated in the UFMylation pathway cascade? Furthermore, with the progress of fundamental biological research, increasing attention has been paid towards the UFM1 system for its potential value in clinical diagnosis and treatment. However, clinical trials focusing on UFMylation are notably absent, and the potential therapeutic targets require in‐depth characterisation, which indicated the need for broader clinical investigation within this field.

**FIGURE 5:**
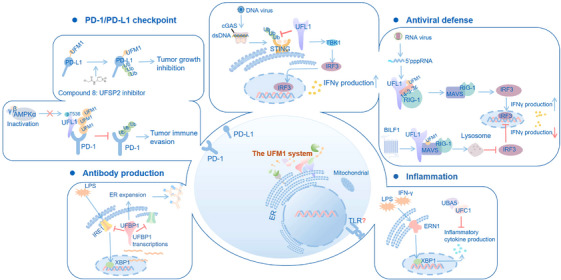
The function of UFMylation in immune response. UFMylation controls adaptive immunity through modulating PD‐1/PD‐L1 checkpoint and B‐cell development and function. UFMylation tags PD‐L1 for proteasome‐mediated degradation.^10^ Depletion of UFM1 components stabilises PD‐L1 through inhibiting its degradation. UFL1 can interact with PD‐1 to promote its UFMylation, antagonising PD‐1 ubiquitination and degradation.^11^ UFL1 ablation in T cells reduces PD‐1 UFMylation, destabilising PD‐1 and enhancing CD8^+^ T‐cell‐mediated anti‐tumour response. The differentiation of plasma B cells stimulates a transcriptional increase of UFBP1 through XBP1s pathway.^12^ Serving as a feedback inhibitor, UFBP1 constrains the activity of sensors IRE1 and PERK, fostering ER expansion and immunoglobulin production. In addition, UFMylation plays critical roles in innate immunity depending on the stimuli and cellular background. UFL1 interacts with STING to prevent its ubiquitination and proteasomal degradation in response to HSV‐1 infection in peritoneal macrophages.^9^ In 293T cells infected with SenV, increased interaction between 14‐3‐3ε and UFL1 leads to UFMylation and subsequent binding to K63‐ubiquitinated RIG‐Ι, enhancing downstream IFN signalling.^7^ During EBV replication in Burkitt lymphoma cell lines, the EBV‐encoded BILF1 mediates MAVS UFMylation, targeting it for lysosomal degradation and potentially disrupting RIG‐I‐MAVS‐driven NLRP3 inflammasome activation and IRF3 responses.^8^ Moreover, the enzymatic activities of UFSP2, UBA5 and UFC1 are required in the negative regulation of macrophage activation mediated by IFN‐γ and LPS.^13^ Transcriptional profiling in cells with deficient UFMylation has revealed an ERN1‐mediated ER stress response, which accounts for the increased sensitivity to pro‐inflammatory stimuli.

The burgeoning interest in UFMylation's role in cellular processes, particularly in immune homeostasis and disorders, represents an exciting avenue for future research.^5^ The review highlights the current understanding of mechanisms and functions of UFMylation across a spectrum of immune responses, including IFN‐γ‐mediated antiviral defence, inflammatory signalling pathways, B‐cell development and function, PD‐1/PD‐L1 checkpoint modulation, the mucosa immunity, and the potential crosstalk between endocrine and immune systems (Figure 5). Despite the notable progress, a number of unsolved issues persist. For example, is the activation and function of other innate immune cells (such as neutrophils, NK cells and mast cells) regulated by the UFM1 system? Are there additional substrates of the UFM1 system involved in immune responses? Given the pivotal role of the association between immune and non‐immune cells, especially within the tumour microenvironment, it is necessary to explore the molecular mechanisms and function of UFMylation‐mediated cell–cell communication.^103^ Importantly, does UFMylation contribute to the modulation of other well‐established checkpoint axes, such as cytotoxic T‐lymphocyte‐associated protein 4, (CTLA4) and T‐cell immunoglobulin and ITIM domain (TIGIT)? If so, what are the underlying molecular mechanisms, and could they be used in cancer immunotherapy as potential targets? Consequently, the regulatory potential of UFMylation in immune responses remains largely undeveloped, awaiting further investigation to uncover its significant contributions to the field.

## AUTHOR CONTRIBUTIONS

*Conceptualisation*: Junzhi Zhou and Yongkang Zou. *Writing of original draft*: Zhengyan Liang and Junzhi Zhou. *Revising the draft*: Zhengyan Liang, Junzhi Zhou, Yafei Cai, Xin‐guang Liu, Zhiwei He, Rongxuan Ning, Xia Kong, Yubin Yan, Yongkang Zou, and Zhaoxiang Wang.

## CONFLICT OF INTEREST STATEMENT

The authors declare they have no conflicts of interest.

## ETHICS STATEMENT

Not applicable.

## Data Availability

Further information may be directed to and will be fulfilled by the lead contact, Junzhi Zhou (zhoujunzhi2013@163.com).
